# Comparison of the Usability of Apple M1 Processors for Various Machine Learning Tasks

**DOI:** 10.3390/s22208005

**Published:** 2022-10-20

**Authors:** David Kasperek, Michal Podpora, Aleksandra Kawala-Sterniuk

**Affiliations:** Department of Computer Science, Opole University of Technology, Proszkowska 76, 45-758 Opole, Poland

**Keywords:** processor architectures, neural processing unit, neural processing cores, NPU benchmark, Apple M1, CoreML, Neural Engine, machine learning, deep learning

## Abstract

In this paper, the authors have compared all of the currently available Apple MacBook Pro laptops, in terms of their usability for basic machine learning research applications (text-based, vision-based, tabular). The paper presents four tests/benchmarks, comparing four Apple Macbook Pro laptop versions: Intel based (i5) and three Apple based (M1, M1 Pro and M1 Max). A script in the Swift programming language was prepared, whose goal was to conduct the training and evaluation process for four machine learning (ML) models. It used the Create ML framework—Apple’s solution dedicated to ML model creation on macOS devices. The training and evaluation processes were performed three times. While running, the script performed measurements of their performance, including the time results. The results were compared with each other in tables, which allowed to compare and discuss the performance of individual devices and the benefits of the specificity of their hardware architectures.

## 1. Introduction

Modern artificial intelligence algorithms make machines better in decision-making applications [1], as well as in interaction-related applications, becoming more and more intuitive for a human user [2,3,4,5]. Computers, with the increase of their computing power, are able to perform many activities much faster than humans [6,7,8,9,10]. The world’s leading research centers are working on algorithms that would allow the machine to compete with the human brain [11,12,13,14]. Adapting (at least partially) intelligent human behavior enables the analysis and appropriate interpretation of data, and thus the advancement in some of the current challenges, including decision-making processes [10,15,16].

Machine learning relies on the analysis of a set of collected inputs to detect patterns, similarities or characteristics [5,17,18,19]. This makes the machine able to ‘learn’ (as the term implies) based on the data received [5,17,20]. Training performed in an iterative form allows one to increase the efficiency and accuracy of predictions [21]. This is because the algorithms that the machine uses in each repetition gather experience, which is then being used for making adjustments [21,22]. Samples of categorized data are also provided for training, thanks to which it is possible to check what result is expected for them. This enables the computer to calculate output values that get closer to the expected value with each iteration. During the learning process, an algorithm is created that enables the machine to process data and, on its basis, also predict values of a specific type [3,19,23,24,25].

The variety of applications is constantly expanded in specialized research centers, both public and private [15,19], and the rapid development of computers enables the processing of more and more data and the use of increasingly complex algorithms. Thanks to machine learning, it became possible to solve complex problems [19,20,21,24,25,26].

The implementation of self-learning algorithms allowed machine learning to be used in solving various tasks [19,20,27,28]. Machine learning models, trained with the use of images and videos, made it possible to extract and identify specific objects, faces and/or persons [19,24,29,30,31]. It is also possible to infer within the time domain, i.e., to generate predictions [31].

### 1.1. Motivation for This Study

Scientists all over the globe are interested in increasing the computational capability of their laptops and desktops in order to increase their productivity and comfort [32,33,34,35]. However, the hardware manufacturers provide only raw parameters (while the computational capability is still strongly dependent on the software frameworks and the operating system), and comparing frameworks is also difficult due to the differences in recommended hardware [36,37].

The most inconvenient aspect of buying new hardware for research work is the fact that the products are often advertised using nonprofessional (marketing) (‘foggy’) expressions/phrases, not related to the actual comfort and computational efficiency during everyday work [38,39].

This is particularly true and important in the case of computers equipped with the Apple silicon chip, while this platform is one of the most tempting options for researchers who want to have hardware acceleration support for their machine learning projects [40,41]. Apple’s M1 chip is equipped with integrated neural cores, which makes this platform worth consideration when ‘updating’ computational hardware [42,43]. However, it is very difficult to find any information on the actual benefits of using a CPU with neural cores, and it is even more difficult to find any comparisons between existing versions of the CPU and between M1 and the Intel based laptops of previous editions.

The authors decided that such a comparison may be very useful, if done with real-world modern machine learning examples, and that the results may be of great value for researchers who are considering the investment in buying a new (possibly neural-core-enabled) laptop.

### 1.2. Scope and Limitations of Study

The research presented within this paper is targeted toward the researcher (laptop buyer) who is trying to make a reasonable choice among Apple’s laptops. The authors’ approach is to clarify and substantiate concrete arguments that present the advantages of particular laptop models in numbers (as opposed to the foggy world of marketing), to enable the possibility to make a fact-based choice on which Apple laptop to buy. The reason for benchmarking only Apple laptops was made upon the fact that M1s have a neural processing unit, and therefore many researchers-laptop buyers may be interested in measuring the real benefits of NPU acceleration in M1s.

This research compares all M1 models against one Intel i5 processor. The i5 is not the most efficient member of its architecture family, but it is the only one available on the market in the new Apple laptops at the moment. Although Apple used to sell i9 based laptops in the past, it failed to fit the preferences of users, possibly due to the increased energy consumption (shorter battery life) and cooling (Apple users are not used to active cooling systems—CPU fans are preferred to be quiet/off), and the use of i9s was discontinued.

The comparison was made only among Apple laptops, and it would be also interesting to see the comparison beyond that limitation. However, it was decided to limit the number of variables to make the research as precise and trustworthy as possible. The authors did not mix operating systems; there was also no disturbance resulting from switching toolboxes, libraries, frameworks and programming languages. The uniform testing conditions were provided on purpose—so that the research would not be accused of being influenced by a poor library choice or questionable framework implementation. It was decided to compare only the current laptops sold by Apple, to assess the real benefits of NPU and the real differences between M1 versions. Of course, the benchmarks of other architectures would be interesting to see in this comparison (including CUDA-enabled platforms), but it would be a completely new challenge (with the difficulty set on making the comparison a just and honest test for different operating systems and environments), which was not in the scope of this paper.

### 1.3. Benchmarking

The benchmarking of computer hardware is performed by using specialized software, designed to stress-test and to show the computational power limitations of the whole system or of its specific components, usually presenting the results in the form of a computation time [44,45,46]. It is very important that the tasks are as close to reality as possible, that is, that they correspond to real use cases in which the equipment will be used. The use of realistic tasks ensures that the measured performance corresponds to ‘real’ applications [47]. ML-related benchmarking is increasingly attracting attention [48,49,50,51].

It is very important to properly analyze and interpret the results after the end of the measurement phase. After collecting data, the difference between the comparative runs of the benchmark executed on the competitive machines is analyzed. The results can be converted into an easy-to-interpret reference scale such as points or ratings, visualized and/or compared to other published results of the same benchmark [52].

## 2. Materials and Methods

According to the title of this paper, the main objective of the research was to assess the usability of M1 based platforms for basic machine learning research tasks (for which a laptop-based environment would be sufficient). The benchmarks and comparisons were made using Swift based ‘Create ML’ machine learning ‘Playground’ project, developed and run in the XCode environment.

Swift is an open-source programming language developed by Apple. Since its release in 2014, the language has continuously evolved to make mobile and desktop programming easy and fast. The syntax is intuitive and allows an interactive programming process. Swift is optimized to perform best on its target devices—platforms developed by Apple. The achieved efficiency is high, because the compiled code is adjusted to the hardware [53].

Xcode is an integrated development environment (IDE) created by Apple for the macOS operating system. It supports various programming languages and includes many tools for the software development process. Using Xcode, the user is able to design user interfaces with dynamical adjustment to the screen size, write code that can take advantage of many frameworks shared by Apple and run the code on physical or virtual Apple devices and many more. Finished applications can be beta-tested by real users using the ‘TestFlight’ program or sent directly to the App Store [54,55].

Xcode Playgrounds are a solution for quick and interactive program development. These simplified IDE versions allow testing the code almost in real time. At the same time, Playgrounds enable the user taking full advantage of the Xcode IDE and its frameworks, without creating large projects. The advantage of easy project sharing and ability to include all datasets in the created playground are the reasons why Xcode Playgrounds were selected for this research.

**Figure 1:**
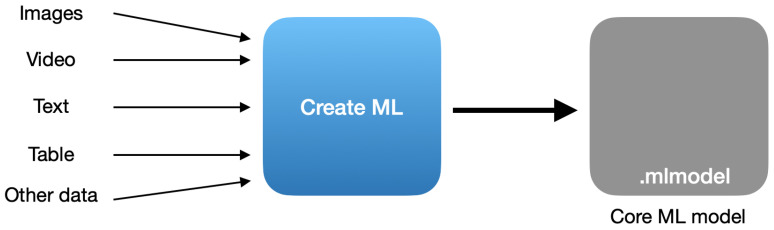
The data used by Create ML for model creation.

The framework ‘Create ML’ was introduced by Apple at the Worldwide Developers Conference in 2018. Its aim is the development of custom machine learning models in a simple way. To train the models, Create ML can analyze various types of inputs, including images, text, tabular data and more. The created models can be implemented in Core ML applications for the Apple platform [23,56]. The inputs and outputs of Create ML are presented in Figure 1.

‘Create ML’ is optimized to perform fast model training on computers that are made by Apple. The model creation process should popularize machine learning technologies in applications for Apple devices [57].

The workflow with ‘Create ML’ involves the following steps: (1) identifying the problem whose kind of action is required from the model, and (2) choosing a training template. ‘Create ML’ ships with a collection of templates including an image classifier, object detection and more. Apple extends this list regularly [58,59].

**Figure 2:**
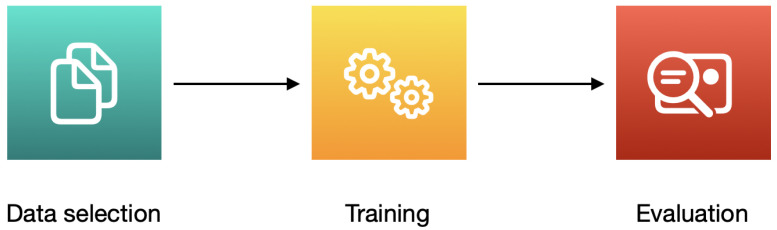
The process of creating a model with Create ML.

The templates are needed to determine the right training algorithm. The training techniques developed by Apple allow to create an efficient machine learning model, even with a small quantity of data. The process performance and memory usage are hardware-optimized. After training, a ‘.mlmodel’ file is created. The machine learning model is saved in it. The file can be implemented in Xcode projects using Core ML [57]. The process of creating a model with Create ML is shown in Figure 2.

‘Create ML’ can be used in two ways: by writing code in the Swift programming language or using a graphical application (graphical user interface, GUI) bundled in Xcode. In both ways, the user is able to create and train custom machine learning models that are ready for implementation in applications [56,59]. In the created project, ‘Create ML’ was used without the GUI.

### 2.1. Model Creation

To create a machine learning model using ‘Create ML’, it is necessary to know what the expected output is, and what data will allow to achieve it. This will enable the selection of an appropriate ‘Create ML’ ‘template’ (that determines the training type) and the preparation of a relevant dataset [57]. The data should be differentiated, in a similar quantity for each of the considered classes, obtained in a similar format, by similar methods and/or sensors. The performance of the model depends on the size of the datasets. The more data used for training, the greater the likelihood of obtaining good results [57,60].

The collected data must be properly segregated and labeled. When processing images, text or audio files, Create ML suggests that the best way is to prepare folders (directories) named after the classes existing within the dataset, and then to place the files in the correct folders. Tabular data can be provided in the form of tabular data files, e.g., ‘.csv’ or ‘.json’, and then converted by Create ML to the MLDataTable type. It is necessary to indicate which columns will be used as ‘features’ and which ones as the ‘target’ columns that are to be returned by the model [57,60].

**Figure 3:**
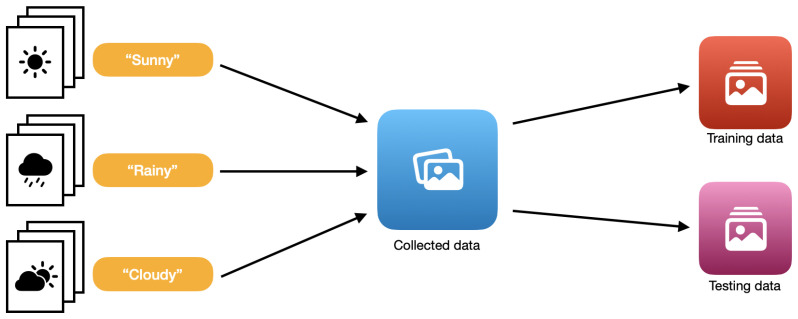
The preparation of datasets for Create ML (image data).

The prepared data should also be divided into two subsets: ‘training data’ on which the training is performed and ‘test data’ used for the evaluation of the created model. Apple recommends randomizing the whole dataset into these two subsets in a ratio of 80:20. This makes it possible to test the model made on data that have not been used for training purposes, which makes the evaluation more accurate. The process of image data preparation for ‘Create ML’ is presented in Figure 3. In the case of tabular data, ‘Create ML’ has a ready function that allows for automatic data division [60,61].

### 2.2. Model Training

**Figure 4:**
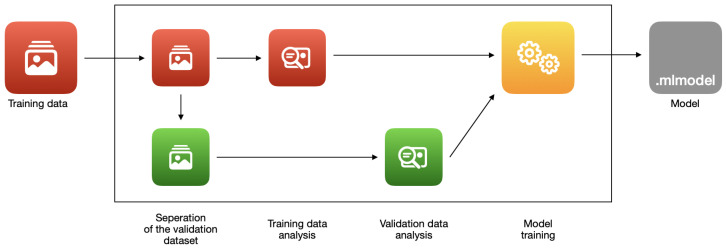
The stages of training an image classifier with ‘Create ML’.

The process of creating a machine learning model in ‘Create ML’ consists of several stages, depending mainly on the selected template. In most cases, the process is similar to the image classifier example shown in Figure 4.

The first step in creating an image classifier is extracting the features from the provided data. The system first extracts a separate set of ‘validation data’ from the ‘training data’ set that is used in the training process. Next, Create ML analyzes the rest of the training data to determine the image features. The same process is repeated on the validation data.

In the next stage, training takes place. ‘Create ML’ uses transfer learning [57], capable of applying an existing model (trained with a dataset relevant to one problem) to a completely new problem. The macOS operating system already has extensive machine learning models [57,62] that were created by Apple. ‘Create ML’ uses their patterns to perform a new training using previously extracted features. This allows to speed up the entire process, increase the accuracy and reduce the size of the models [57].

**Figure 5:**
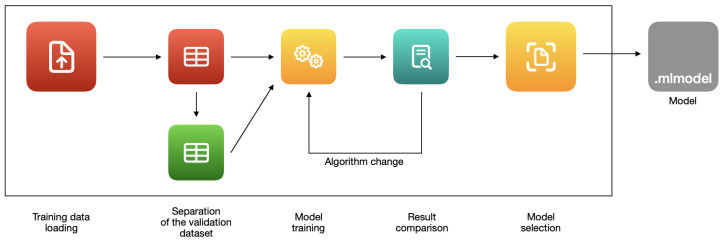
The stages of training a tabular classifier with ‘Create ML’.

The case training a tabular data model is presented in Figure 5. At the beginning, the data are read and then the validation subset is extracted. If the user has chosen a specific algorithm to be used for training, it is used. However, there is also a possibility of an automatic selection of the algorithm. In this case, ‘Create ML’ performs the model training using various algorithms with parameters selected by itself and then compares their results. The process continues until the optimal solution is found.

### 2.3. Model Evaluation

After completing the training, it is possible to evaluate the model. For this purpose, the test subset of the dataset is used. ‘Create ML’ first repeats the process of analyzing the received data, and then passes the data to the model. In the next step, the feedback responses from the model are confronted with the ground truth (labeled testing dataset). Evaluation results are usually lower than training and validation results because tests are performed on this part of the dataset, which has not been involved at the training stage. This allows for a better presentation of the situation of a real application of the model [60].

### 2.4. Model Export

After training, a ‘.mlmodel’ file is created containing the saved machine learning model. This file can be imported directly into Xcode projects and implemented using ‘Core ML’. It is also possible to save the file in a selected location on the disk or to share it using file sharing [57].

### 2.5. Datasets Used within This Research

To perform this research about the machine learning model creation time, three model types were selected:

- Image classifier;
- Tabular classifier;
- Tabular regressor.

Two image datasets and two tabular datasets were used in this work.

#### 2.5.1. ClassifierData Dataset (Photos)

**Figure 6:**
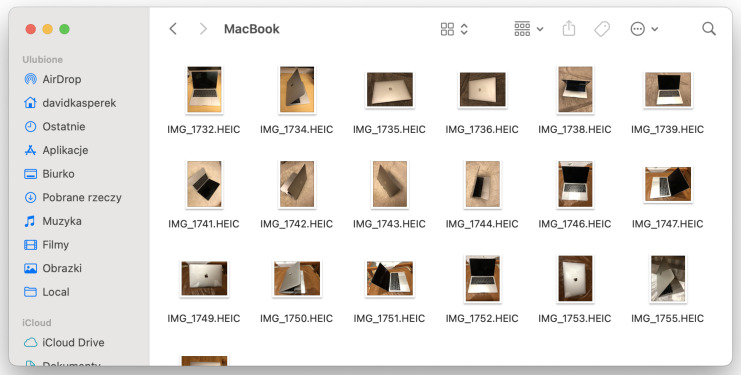
Photos of category ‘MacBook’ form the training data in ‘ClassifierData’.

The dataset ‘ClassifierData’ contains images of electronic devices divided in 4 categories: ‘iPhone’, ‘MacBook’, ‘Apple Watch’ and ‘AirPods’. Each category counts 25 photos captured with an iPhone X. The pictures present the devices from different angles and in various light conditions. A sample view of the dataset is shown in Figure 6.

**Figure 7:**
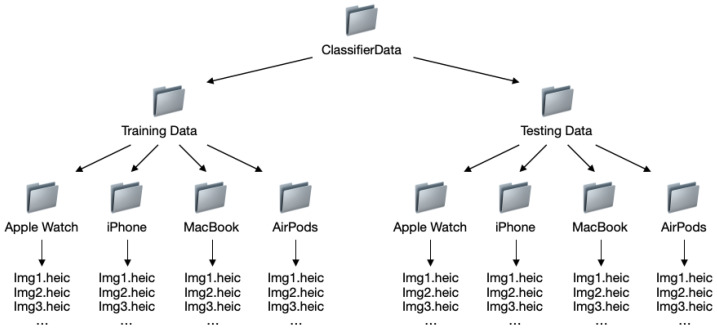
The directory structure in ‘ClassifierData’.

The dataset is divided into two parts: training data and testing data, split with a ratio of 4:1. The directory structure of the dataset complies with Apple’s ‘Create ML’ guidelines [60] and is presented in Figure 7. The size of the ‘ClassifierData’ dataset is 121.7 MB.

#### 2.5.2. Animals Dataset (Kaggle)

An image dataset called ‘Animals’ is available on the Kaggle platform, within the project named ‘*Animals Detection Images Dataset. Collection of wild animal species with annotations*’ [63]. The collection was made available to the public domain under a Creative Commons license.

**Figure 8:**
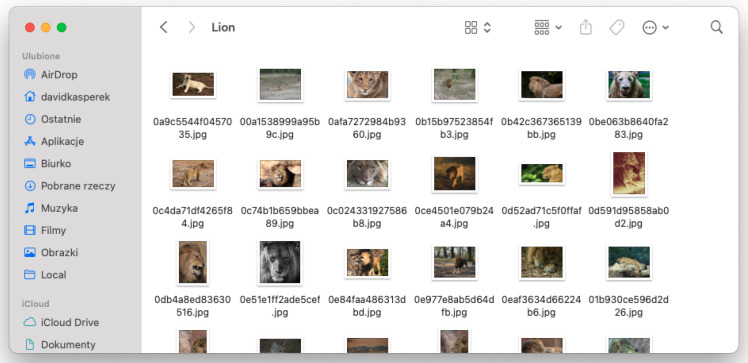
Photos of category ‘Lion’ in the ‘Animals’ training dataset.

The ‘Animals’ dataset is divided into two subsets for training and for testing. In each of them there are photos in 80 different categories. They include various types of animals such as dogs, cats, lions, tigers and many others. A sample view of the dataset is presented in Figure 8. The size of the entire dataset is 9.58 GB. This research was conducted by using the following quantities for the training, validation and testing—21,468, 1098 and 6505, respectively. Label files were removed from each category as they are not used for the ‘Create ML’ image classifier.

#### 2.5.3. PaymentFraud Dataset (Kaggle)

The ‘PaymentFraud’ dataset refers to the ‘*Online Payments Fraud Detection dataset’. Online payment fraud big dataset for testing and practice purpose*’, from the Kaggle platform [64]. The dataset includes 5,080,807 entries, divided into two classes, each entry described by eight features. Its author is the user ‘Rupak Roy’. The collection was made available under a Creative Commons–attribution–noncommercial license.

**Figure 9:**
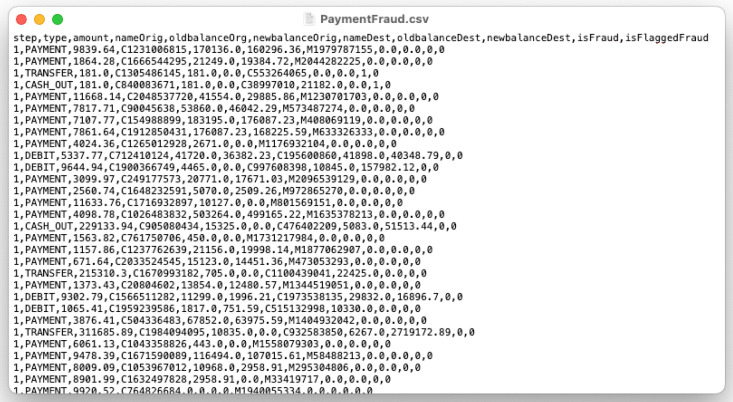
An example view of the ‘PaymentFraud.csv’ file.

The set consists of a ‘.csv’ file containing data about online transactions, including, inter alia, the sender’s and recipient’s account balances, amount and type of transaction. Each entry also contains an ‘isFraud’ label, which describes whether the transaction was normal (0) or was a fraud (1). A view of the dataset is shown in Figure 9. The size of the ‘PaymentFraud.csv’ file is 493.5 MB.

#### 2.5.4. SteamReviews Dataset (Kaggle)

The ‘SteamReviews’ dataset contains approximately 21 million user reviews of games available on the Steam platform. It was uploaded to the Kaggle platform by ‘Marko M.’ under the name ‘*Steam Reviews dataset 2021. Large collection of reviews of Steam games*’ [65]. The dataset is available under the GNU GPL 2 license.

**Figure 10:**
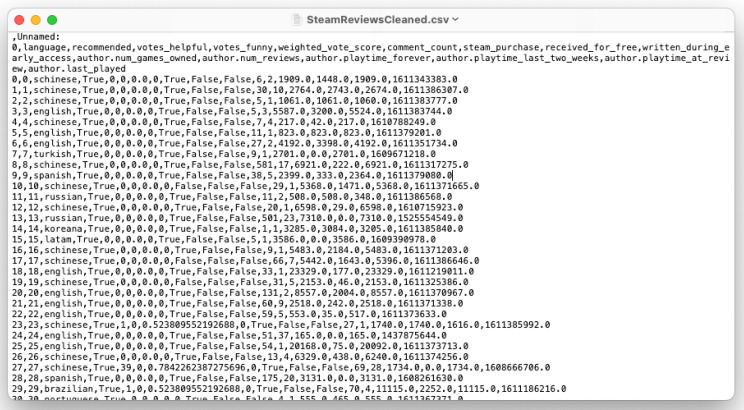
A preview of the ‘SteamReviewsCleaned.csv’ file.

In order to adapt the dataset for use in ‘Create ML’, a cleanup was performed. The Python programming language was chosen for its ease of programming and adaptation to working with data. Using the ‘Pandas’ package, the contents of the ‘.csv’ file were loaded into the ‘DataFrame’ format. Then the data was cleaned, by removing some of the columns, including the comment text, author, creation date and others. Finally, the cleaned dataset was saved to a 2.15 GB file ‘SteamReviewsCleaned.csv’. A sample view of the file is presented in Figure 10.

### 2.6. Choosing Appropriate Classifiers for a Particular Dataset

As part of the study, the training and evaluation of 4 machine learning models were carried out. The following classifier were used for each of the four datasets:

- ClassifierData—image classifier;
- Animals—image classifier;
- PaymentFraud—tabular classifier;
- SteamReviews—tabular regressor.

### 2.7. Models Creation

The models were created using functions written in the Swift programming language. The functions are described below.

Each image classifier was created in a function called ‘createImageClassifier’. The training dataset was passed using the ‘MLImageClassifier.DataSource’ type. At the beginning, the actual timestamp (with nanosecond accuracy) was saved. Next, the ‘Create ML’ process of training an image classifier was started using the ‘MLImageClassifier’ function. The whole process was documented/logged in the console. When the training was done, another timestamp was saved and the total training time was calculated. The function returned the created model and time value in seconds.

Implementing the use of the ‘createTabularClassifier’ function was similar to the process of creating an image classifier. The only differences were that the data type was different—‘MLDataTable’, and the Create ML function ‘MLClassifier’ took not only the training data but also required indicating the target column for classification. The time of the training was measured and returned (in seconds), as well as the created model.

A function called ‘createTabularRegressor’ was used to create a tabular regressor. It took in the same arguments as the ‘createTabularClassifier’ function. However, the missing data needed to be removed from the ‘MLDataTable’ using the ‘data.dropMissing()’ command. The ‘MLLinearRegressor’ function performed a Create ML training of a tabular regressor model.

### 2.8. Model Testing

Each model was tested using a dedicated function. The time of the Create ML testing process was also measured and the results acquired.

The ‘testImageClassifier’ function took the testing dataset as an argument in the form of a ‘MLImageClassifier.DataSource’ type, as well as the created machine learning model. Firstly, the initial timestamp was acquired. Then, the Create ML testing process was performed, and the percentage of correctness was calculated based on the result returned. The function calculated the total time of the testing by subtracting the initial timestamp from a final timestamp. The evaluation of a tabular classifier was almost the same, except for the model type (‘MLClassifier’) and the testing data type (‘MLDataTable’).

To perform a test of a trained tabular regressor, the ‘testTabularRegressor’ function was used. The data type of the model was ‘MLLinearRegressor’ and the testing dataset in the form of ‘MLDataTable’. After the evaluation, the maximum error and RMSE (root-mean-square error) were returned. The function also measured the time of the testing process.

### 2.9. Parent Functions of Creating Models

The process of training and testing machine learning models was controlled by special functions, created for each ‘Create ML’ template type. The functions loaded the datasets, called the subfunctions described before and presented the results in console. This reduced the required amount of code, because in the case of the image classifier, functions were universal—they could be used with various datasets.

In the ‘createImageModel’ function, the image classifier was created and evaluated. Firstly, the paths to the training and testing data were created, based on the dataset name passed. Then, the data was loaded into the ‘MLImageClassifier.DataSource’ type, and the directory structure of ‘labeledDirectories’ type was declared. The model was created using the ‘createImageClassifier’ subfunction. The training and validation accuracies were calculated from the result. Then, the model evaluation was performed using the ‘testImageClassifier’ subfunction. All results were printed to the console.

The tabular data was handled in a similar way. To assess the tabular classifier, a function ‘createTabularClassifierModel’ was used. At the beginning, a ‘.csv’ file with the dataset was opened and loaded into the ‘MLDataTable’ type. Next, the feature columns were declared and a new ‘MLDataTable’ was created using the feature columns only. The new table was then randomly split into training and testing datasets, in compliance with the recommended 8:2 proportion. To create a tabular classifier, the ‘createTabularClassifier’ subfunction was used. The subfunction took the training data and a target column as arguments. The created model and testing dataset were passed to the ‘TestTabularClassifier’ subfunction. The obtained results were presented in the console. The tabular regressor research process differed from the classifier by the obtained data types and datasets.

### 2.10. Measurement of Execution Time

In order to obtain a greater reliability of the results when measuring the time of creating the models, the study was performed in a loop. All measurements were made three times. The total run time of each iteration of the loop, the development process and the model testing process were measured. All information about the script running status was reported in the console on an ongoing basis.

### 2.11. Hardware Used

The research was carried out on 4 Apple MacBook Pro computers. Each device was running macOS Monterey version 12.4 [66], with Xcode version 13.4 installed.

The four above-mentioned notebooks were chosen to reflect the buyer’s options when buying a new laptop. The main difference was the CPU version (and architecture): Intel i5 (8 GB), Apple M1 (8 GB), Apple M1 Pro (16 GB) and Apple M1 Max (32 GB).

The first system compared was the Apple MacBook Pro 2016. It is the only one of the compared computers operating on the Intel architecture. It has a dual-core Intel Core i5 2.9 GHz processor, 8 GB of RAM and an integrated graphics chip Intel Iris Graphics 500, equipped with 1536 MB. This computer is referred to as ‘i5’ in this work.

The next tested device was the 202 MacBook Pro. It is a computer with the first Apple M1 integrated chip, based on the ARM architecture. It has 8 computing cores: 4 high-performance and 4 energy-saving. The chipset also includes an 8-core GPU (graphical processing unit) and a 16-core Neural Engine—Apple’s custom NPU (neural processing unit), accelerating machine learning computations [67]. The computer also has 8 GB of RAM. It is referred to as ‘M1’ in this research.

Another computer on which the tests were performed was the MacBook Pro 2021 with the M1 Pro chip. This is a higher option on the Apple chipset, featuring an 8-core CPU and a 14-core GPU. The main difference between the M1 and the M1 Pro systems is the division of the functionality of the processor cores—the M1 Pro uses 6 cores as high-performance and 2 as energy-saving ones. The memory interface is also improved [68]. The M1 Pro also features 16-core Neural Engine [68]. The computer is equipped with 16 GB of RAM. It is referred to as ‘M1 Pro’ for easy recognition.

The fourth computer tested was the MacBook Pro 2021 with the M1 Max chip. It is a processor equipped with 10 CPU cores (8 high-performance and 2 energy-saving), 24-core GPU and 16-core Neural Engine and 32 GB of RAM. According to [68], the bandwidth of the system is improved compared to the M1 Pro [68], as well as the built-in memory [68]. It is referred to as ‘M1 Max’.

## 3. Results

Two groups of measurements were carried out as part of this research:

- The comparison of execution time between standard boot mode and the special safe boot mode—since it is possible to boot a computer with macOS in normal boot mode or in the so-called safe mode (which could have produced significantly different results), both scenarios were tested by running a series of measurements of the execution time using the exact same system;
- A comparative study of the creation time of the machine learning models mentioned in the previous section, on all four machines.

### 3.1. Benchmark of the Influence of the macOS Boot Mode

A comparative study of the two boot modes was carried out using the ‘i5’ MacBook Pro. The test was performed by first putting the computer into safe mode, executing the script, and then back to normal boot, and executing the script. The device was connected to the power supply all the time in order to avoid any restrictions resulting from possible energy saving.

In the case of the ‘ClassifierData’ dataset, the total creation and testing time for the machine learning model was lower for the safe mode, although the shortest model total training time was obtained in safe mode. For each of the runs, the training accuracy and validation accuracy were both equal to 100%. During the runs, different numbers of training iterations were obtained—in the case of the safe mode it was 14, 12 and 12, and for the normal boot mode, it was 13 in each case. The total training time was shorter by more than 10 s in the safe mode, which had an impact on the overall evaluation results. However, in the testing process, each safe-mode model made one error, which translated into a decrease in accuracy to 95.83% compared to 100% obtained in the normal boot mode. The comparison of the averaged measurement results for the ‘ClassifierData’ dataset is presented in Table 1.

The analysis of the ‘Animals’ dataset showed significant differences between the safe and normal boot modes. Their averaged results are compared in Table 2. The ‘training data—analysis time’ was almost three times longer in the safe mode compared to the normal boot mode. Each training session consisted of 24 iterations as this was the maximum value in ‘Create ML’ by default. However, in the case of the ‘model training—total time’, the time results were comparable. This might mean that the CPU access time/mode was the same in both boot modes of the system, and the significance of the differences in the training time (and validation time, thus also in the total time) resulted from the limitations of the safe mode, for example from the disabled acceleration. The correctness scores (the accuracy) suggested that the models created were nearly identical.

In the case of examining the tabular classifier using the data from the ‘PaymentFraud’ dataset, very similar results were obtained. The total model creation time was slightly shorter in the safe mode. The training accuracy in both modes was identical, and the validation accuracy differed only by 0.01 percentage points. This suggested that the models created might be extremely similar. The comparison of the averaged results of examining the ‘PaymentFraud’ dataset is presented in Table 3.

In the linear regressor analysis, all results were very similar. The total model creation time was 1.8% higher in the normal boot mode than in the safe mode. The same results were obtained for the highest error values and root-mean-square errors. The results are compared in Table 4.

The execution time for each iteration of the loop was significantly faster with the normal mode. In all cases, the iteration time in the safe boot mode exceeded 220% of the normal boot mode’s iteration time. The total measurement time in the normal mode was 6177 s (over 1 h and 42 min), whereas in case of the safe boot mode the measurement time was more than double (over 3 h and 52 min). The comparison of exemplary iteration test times in safe and normal boot mode are presented in Table 5.

As a result of a comparative study, no significant benefits were found for running machine learning tasks in the safe boot mode (despite fewer processes running in it). Slightly shorter safe boot mode total processing times were observed for the ‘ClassifierData’, ‘PaymentFraud’ and ‘SteamReviewsCleaned’ datasets, but significantly longer data processing time for the ‘Animals’ dataset outweighed the overall study result. For this reason, it was decided to abandon the idea of running tests in the safe boot mode and perform the remaining tests using the normal macOS boot mode.

### 3.2. Processor Architecture Benchmark

As part of the research, three measurements of training time and model testing time allowed us to assess and compare the efficiency of the machines for typical ML use cases.

#### 3.2.1. Running the Benchmark

The test was carried out on each of the computers. The machines were connected to the power supply and booted up (into the normal macOS boot mode). The file ‘Benchmarker_Playground.playground’ was copied onto each computer’s hard drive. The file was loaded into the Xcode environment and executed. The computers were not processing any additional tasks/software during the study to avoid additional CPU load. After the program had finished, the console output was saved in a ‘.txt’ file.

**Figure 11:**
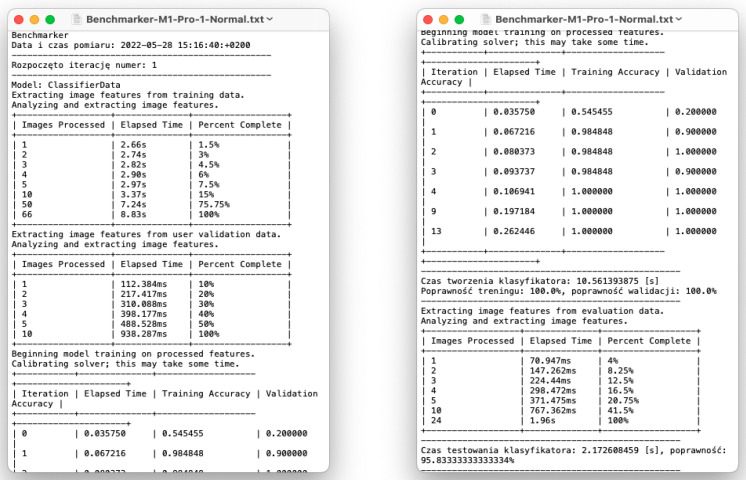
Fragments of the output for the measurement results of the ‘M1 Pro’ computer, performed using the ‘ClassifierData’ dataset. Left—beginning of the measurement; right—during the measurement process.

Two exemplary screenshots with benchmark report output from the test of the ‘M1 Pro’ machine are presented below in Figure 11.

#### 3.2.2. The Results of the Benchmark

Table 6 presents the accuracy and time results of the process of creating and testing the model performed using the ‘ClassifierData’ dataset.

While working with the ‘ClassifierData’ dataset, the shortest average data analysis times were observed for the ‘M1’ computer. Its average ‘training data—analysis time’ was more than nine times shorter than that of the ‘i5’ computer. Computers with the ‘M1 Pro’ and ‘M1 Max’ chipsets achieved similar data analysis times. The longest ‘model training—total time’ was obtained by the computer with an i5 processor, a bit shorter on a device with the ‘M1 Max’ chip, and the best results were achieved by ‘M1’ and ‘M1 Pro’. Despite slight variations in the number of iterations per training, their impact on the final result did not seem to be substantial. All models achieved 100% accuracy for training as well as for validation. The ‘i5’ computer model creation time was 8.44 times longer than of the ‘M1’ computer. Despite this, it achieved 100% for the evaluation accuracy, unlike the other systems.

For the ‘i5’, ‘M1’ and ‘M1 Pro’ computers, it was observed that the training always took longer during the first iteration of the measurement loop (similarly when using the safe mode analyzed in Section 3.1). The validation and testing stages of the measurement loop did not have such clear disproportions for the first iteration as the training stage. Data analysis times in iterations are presented in Table 7.

Table 8 shows average test results for the ‘Animals’ dataset. The shortest mean data analysis times were observed on the ‘M1 Pro’ computer. All computers with Apple chipsets (’M1’, ‘M1 Pro’ and ‘M1 Max’) achieved similar data analysis times. The ‘i5’ computer clearly stayed behind. The training data analysis time was 917 s (over 15 min) longer compared to that of the ‘M1 Pro’. The model training process for the image classifier was the fastest on the ‘M1 Pro’ computer. Its time was only 18% of the result of ‘i5’. The ‘M1 Max’ and the ‘M1 Pro’ computers had similar results. The ‘model training—total time’ lasted 86% longer on the ‘M1’ than on ‘M1 Pro’ computers. All computers achieved a similar accuracy in training, validation and testing. The ‘M1 Pro’ computer had the shortest total model creation and testing times, while the ‘i5’ had the longest. In the case of model creation, the difference was over 1171 s (almost 20 min).

As for the ‘PaymentFraud’ tabular dataset, the ‘M1 Max’ computer achieved the shortest average initial data processing time—processing of 493.5 MB took on average 2.090 s. The second shortest result belonged to the ‘M1 Pro’ computer, and the third to the ‘M1’ computer. All computers with the Apple chipset achieved similar initial data processing times. The ‘i5’ computer needed more than three times longer. All computers achieved the same training accuracy and almost identical validation accuracy, in all cases close to 100%. As for the total model creation time, the disproportions were not as impressive as for the previous datasets. The shortest average time was obtained by the ‘M1’ computer, and the longest by the ‘i5’ computer. The difference between them was 94.803 s (the ‘i5’ computer processing time took 1.8 times longer than the ‘M1’ computer). The ‘M1 Pro’ and ‘M1 Max’ computers had similar model creation times. The evaluation of the created tabular classifiers showed that all models achieved 99.96% correctness in testing. The ‘M1 Max’ computer performed the fastest during model evaluation, and the ‘i5’ computer performed the longest. Average test results are presented in Table 9.

The analysis of the ‘SteamReviewsCleaned’ tabular dataset is presented in Table 10. It was the first one to give arguments for the superiority of the ‘M1 Max’ computer. In all timing categories, the ‘M1 Max’ computer achieved the shortest average scores. The ‘M1 Pro’ and ‘M1 Max’ computers’ similarity in results was clearly visible, although the ‘M1 Pro’ computer was almost able to keep up with the ‘M1 Max’ computer. The initial data processing time of the ‘i5’ computer was 3.58 times longer than that of the ‘M1 Max’ computer. The difference in the ‘total model creation time’ between the ‘M1 Max’ and ‘M1 Pro’, ‘M1 Max’ and ‘M1’ and ‘M1 Max’ and ‘i5’ computers was 1.846 s, 3.944 s and 36.698 s, respectively. All computers obtained identical results for the maximum error value and root-mean-square errors for training, validation and evaluation. The evaluation was also the fastest on the ‘M1 Max’ computer. 

It took an average of 431.150 s for the ‘M1 Pro’ computer to iterate over the benchmark loop of the ‘Benchmarker_Playground’ program (see Table 11). The ‘M1 Max’ computer achieved a similar average result, longer by only 1.821 s. The ‘M1’ computer iterated on average in 454.539 s. The ‘i5’ computer obtained the longest result, with an average iteration time of 2058.969 s.

Of all of the measurement iterations, the fastest single iteration time was achieved by the ‘M1 Max’ computer, in 423.963 s. It is worth noting that the fastest iteration of the ‘M1’ computer (446.181 s) was still longer than the fastest iteration of the ‘M1 Max’ computer (445.702 s).

The program also measured the total running time. The best result was obtained by the ‘M1 Pro’ computer, performing all tests in 1293.450 s (over 21 min and 33 s). The ‘M1 Max’ computer had a similar result—1298.913 s. The ‘M1’ computer completed all tasks in approximately 5% longer than the ‘M1 Pro’ computer. The ‘i5’ computer needed 6176.917 s (almost 1 h and 43 min).

## 4. Discussion

The research included a comparative analysis of the computational performance of four currently available Apple laptop processor models in machine learning applications performed using ‘CreateML’—a machine learning tool designed by Apple. As Create ML is able to create the same machine learning models, regardless of the hardware it runs on, all models obtained satisfactory results, which confirmed their usability and applicability in the prepared use cases.

The obtained results of the processing time measurements clearly showed that the ‘M1 Pro’ and ‘M1 Max’ computers were best suited for ‘CreateML’ machine learning applications. Older computers, although technically weaker, were still capable of making useful machine learning models, although it took considerably more time. The use of three publicly available datasets in the work enabled the research to be repeated and the results to be compared.

On the basis of the obtained results the following conclusions can be drawn:

- The computer equipped with the ‘M1’ chipset was the best one at creating models from smaller datasets;
- The ‘M1 Pro’ and ‘M1 Max’ systems usually achieved similar results;
- The ‘M1 Max’ was the best one at processing tabular data;
- The advantage of the ‘M1 Max’ system over the ‘M1 Pro’ grew with the size of the tabular dataset;
- Despite the longest training and validation times, the ‘i5’ computer created similar or identical machine learning models, and in the case of the ‘ClassifierData’ dataset, it even achieved the highest accuracy;
- The number of iterations of the training process in ‘Create ML’ did not translate directly into the correctness obtained;
- The ‘training data—analysis time’ and validation had no direct effect on the ‘model training—total time’ of the model;
- Despite the use of the same Neural Engine accelerator (NPU) in the ‘M1’, ‘M1 Pro’ and ‘M1 Max’ systems, their training times were different, which suggests that the training process was also influenced by other parameters of the microcircuit and/or system;
- In all of the time results of the analysis or data processing, there was a similarity in the results of Apple’s M1 based chipsets;
- The evaluation confirmed that the prepared models were almost identical or the same (in the case of tabular sets), for each model;
- The ‘evaluation accuracy’ was usually lower than the ‘training accuracy’ and ‘validation accuracy’.

It is possible to extend the ‘Benchmarker’ program with the possibility to use new data sets. Its methods and functions are already prepared to allow for the easy use of new data. This would allow us to check how the processors/systems deal with datasets of different sizes and properties. Another new research opportunity is to perform the benchmarks on other computers. This would allow the results to be confronted with other macOS-compatible hardware options (including iMac or Mac Pro) and to check their machine learning performance.

It would also be interesting to analyze DL-based image processing models with image outputs, as they require much larger computational resources; however, the graphical output would make it much more challenging to determine the correctness and compare the efficiency among the tested platforms.

## 5. Summary

As a result of the tests (proposed and run as a part of this research), the following practical reflections can be stated:

- The ‘i5’ computer, despite that it is not the latest version of the Apple Macbook Pro, is able to achieve similar or identical machine learning models as the new versions, although it needs more time;
- The advantage of the Neural Engine (NPU accelerator) is clearly visible, giving a huge boost on all M1 based machines tested (remarkably reducing the required processing time);
- The processing time differences between the ‘M1’, ‘M1 Pro’, and ‘M1 Max’ systems in machine learning applications does not seem to make a huge difference.

All of the performed tests clearly showed the benefits of hardware acceleration offered by the NPU included within the design of the Apple M1 processor family. The authors of this work hope that the NPUs not only will be upgraded, enhanced and empowered in future versions of this particular processor family, but that they will also become a standard feature in other/all new processor architectures.

## Figures and Tables

**Table 1:** Comparison of the averaged measurement results for the ‘ClassifierData’ dataset, executed using the safe and normal boot mode.

	Safe Mode	Normal Mode
Training data—analysis time (s)	16.43	26.64
Validation data—analysis time (s)	2.47	2.91
Model training—total time (s)	0.31	0.41
Model training—number of iterations	12.67	13
Training accuracy	100%	100%
Validation accuracy	100%	100%
**Total model creation time (s)**	**20.15**	**31.70**
Evaluation data—analysis time (s)	5.87	8.63
Evaluation accuracy	95.83%	100%
**Total model evaluation time (s)**	**6.25**	**8.99**

**Table 2:** Comparison of the averaged measurement results of the ‘Animals’ dataset for the safe and normal boot mode.

	Safe Mode	Normal Mode
Training data—analysis time (s)	3002	1055
Validation data—analysis time (s)	151	54
Model training—total time (s)	224.62	216.05
Training accuracy	88.15%	88.19%
Validation accuracy	87.10%	86.37%
**Total model creation time (s)**	**3425.23**	**1370.42**
Evaluation data—analysis time (s)	879	319
Evaluation accuracy	84.83%	84.68%
**Total model evaluation time (s)**	**894.15**	**332.66**

**Table 3:** Comparison of the averaged measurement results of the ‘PaymentFraud’ dataset for the safe and normal boot mode.

	Safe Mode	Normal Mode
Data processing time (s)	6.65	6.83
Training accuracy	99.96%	99.96%
Validation accuracy	99.96%	99.97%
**Total model creation time (s)**	**203.54**	**213.56**
Evaluation accuracy	99.96%	99.96%
**Total model evaluation time (s)**	**3.67**	**3.73**

**Table 4:** Comparison of the averaged measurement results of the ‘SteamReviewsCleaned’ dataset for the safe and normal boot mode.

	Safe Mode	Normal Mode
Data processing time (s)	29.129	29.608
Training—maximum error	2,428,429	2,428,429
Training—root-mean-square error	19,627	19,627
Validation—maximum error	1,841,631	1,841,631
Validation—root-mean-square error	19,834	19,834
**Total model creation time (s)**	**48.885**	**49.776**
Evaluation—maximum error	2,653,823	2,653,823
Evaluation—root-mean-square error	19,605	19,605
**Total model evaluation time (s)**	**6.778**	**6.859**

**Table 5:** Comparison of exemplary iteration test times in safe and normal boot mode.

	Safe Mode	Normal Mode
First iteration time (s)	4819	2101
Second iteration time (s)	4538	2036
Third iteration time (s)	4590	2040
**Total time (s)**	**13,947**	**6177**

**Table 6:** Average results for the test performed using the ‘ClassifierData’ dataset.

	i5	M1	M1 Pro	M1 Max
Training data—analysis time (s)	26.640	2.887	6.957	7.083
Validation data—analysis time (s)	2.910	0.334	0.921	0.888
Model training—total time (s)	0.412	0.243	0.217	0.333
Model training—number of iterations	13	11	12.3	14.3
Training accuracy	100%	100%	100%	100%
Validation accuracy	100%	100%	100%	100%
**Total model creation time (s)**	**31.697**	**4.111**	**8.571**	**8.773**
Evaluation data—analysis time (s)	8.633	0.821	1.933	2.053
Evaluation accuracy	100%	95.83%	95.83%	97.22%
**Total model evaluation time (s)**	**8.989**	**0.997**	**2.138**	**2.287**

**Table 7:** Comparison of data analysis times for the ‘ClassifierData’ dataset.

Processor	Iteration	Training Data (s)	Validation Data (s)	Testing Data (s)
i5	I	32.10	3.59	8.53
	II	23.42	1.46	8.53
	III	24.40	3.68	8.84
M1	I	4.05	0.347	0.879
	II	2.20	0.326	0.802
	III	2.41	0.330	0.783
M1 Pro	I	8.83	0.938	1.96
	II	5.88	0.898	1.89
	III	6.16	0.928	1.95
M1 Max	I	7.34	0.67	1.61
	II	8.36	1.15	2.79
	III	5.55	0.838	1.76

**Table 8:** Average test results for the ‘Animals’ dataset.

	i5	M1	M1 Pro	M1 Max
Training data—analysis time (s)	1054.667	142.333	137.667	142.667
Validation data—analysis time (s)	54.387	7.233	7.160	7.347
Model training—total time (s)	216.043	75.230	40.423	42.720
Training accuracy	88.19%	88.17%	87.91%	88.15%
Validation accuracy	86.37%	86.89%	86.12%	87.71%
**Total model creation time (s)**	**1370.423**	**243.957**	**198.987**	**206.137**
Evaluation data—analysis time (s)	319.000	44.823	41.977	43.857
Evaluation accuracy	84.68%	84.94	84.94%	85.11%
**Total model evaluation time (s)**	**332.660**	**51.280**	**46.590**	**48.730**

**Table 9:** Average test results for the ‘PaymentFraud’ dataset.

	i5	M1	M1 Pro	M1 Max
Data processing time (s)	6.831	2.343	2.160	2.090
Training accuracy	99.96%	99.96%	99.96%	99.96%
Validation accuracy	99.97%	99.95%	99.97%	99.96%
**Total model creation time (s)**	**213.561**	**118.758**	**143.456**	**137.789**
Evaluation accuracy	99.96%	99.96%	99.96%	99.96%
**Total model evaluation time (s)**	**3.727**	**1.504**	**1.298**	**1.272**

**Table 10:** Average test results for the ‘SteamReviewsCleaned’ dataset.

	i5	M1	M1 Pro	M1 Max
Data processing time (s)	29.608	9.927	8.347	8.268
Training—maximum error	2,428,429	2,428,429	2,428,429	2,428,429
Training—root-mean-square error	19,627	19,627	19,627	19,627
Validation—maximum error	1,841,631	1,841,631	1,841,631	1,841,631
Validation—root-mean-square error	19,834	19,834	19,834	19,834
**Total model creation time (s)**	**49.776**	**17.022**	**14.924**	**13.078**
Evaluation—maximum error	2,653,823	2,653,823	2,653,823	2,653,823
Evaluation—root-mean-square error	19,605	19,605	19,605	19,605
**Total model evaluation time (s)**	**6.859**	**2.348**	**1.732**	**1.648**

**Table 11:** Average iteration times of the ‘Benchmarker_Playground.playground’ program.

	i5	M1	M1 Pro	M1 Max
Average iteration time (s)	2058.969	454.539	431.150	432.971
Fastest iteration (s)	2035.695	446.181	428.403	423.963
Longest iteration (s)	2101.150	462.364	436.282	445.702
**Total measurement time (s)**	**6176.917**	**1363.620**	**1293.450**	**1298.913**

## Data Availability

The Swift code of the ‘Benchmarker’ project is available on GitHub at https://github.com/dKasperek/Benchmarker (uploaded/accessed on 25 August 2022). By default, the code runs three times. In each iteration four machine learning models are created (the used datasets are ‘ClassifierData’—available in the GitHub project, ‘Animals’ [63], ‘PaymentFraud’ [64] and ‘SteamReviews’ [65]). Datasets must be imported to the ‘Resources’ folder inside the Xcode project.
